# Case Report: Lethal neonatal form of CPT II deficiency in consecutive pregnancies: fetal-neonatal characteristics, biochemical and molecular review

**DOI:** 10.3389/fped.2025.1648282

**Published:** 2025-11-18

**Authors:** Y. Y. Tan, K. G. Tewani, A. J. Anand, C. X. Kong, V. S. Rajadurai, S. Chandran

**Affiliations:** 1 Department of Neonatology, KK Women’s and Children’s Hospital, Singapore, Singapore; 2 Pediatric Obstetrics & Gynecology Clinical Program, Duke NUS Medical School, Singapore, Singapore; 3 Perinatal Palliative Care, KK Women’s and Children’s Hospital, Singapore, Singapore; 4 Pediatric Academic Clinical Program, Lee Kong Chian School of Medicine, Singapore, Singapore; 5 Pediatric Academic Clinical Program, Yong Loo Lin School of Medicine, Singapore, Singapore; 6 Pediatric Academic Clinical Program, Duke NUS Medical School, Singapore, Singapore

**Keywords:** CPT II deficiency, carnitine, fatty acid oxidation, polycystic kidneys, ventriculomegaly

## Abstract

Carnitine palmitoyltransferase II (CPT II) deficiency is a rare inherited disorder of mitochondrial oxidation of long-chain fatty acids (LCFA). Carnitine is the sole carrier of LCFA, which is transferred to the cellular mitochondria for *β*-oxidation; CPT II plays a vital role in this process. Three phenotypic forms of CPT II deficiency exist: lethal neonatal (LNF), severe infantile hepato-cardio-muscular, and mild adult myopathic forms. The LNF is the most severe type of CPT II deficiency. Management should be guided by shared decision-making with parents, taking into account the severity of the disease, the goals of care, and the quality of life for both the patient and their family. We present a consanguineous couple with two siblings in consecutive pregnancies with LNF of CPT II deficiency who exhibited similar symptoms, both antenatally and postnatally. Antenatal assessments revealed cardiomegaly, ventriculomegaly, and polycystic kidneys. The first sibling received all supportive measures, including extracorporeal life support, but succumbed. Parents, counseled antenatally by the perinatal palliative care team, opted for comfort care for the second sibling, who passed away on day 3 of life. Cardiac, renal, and cerebral malformations were consistent in fetal and neonatal ultrasound scans of both siblings, who had biochemical and molecular diagnoses, confirming CPT II deficiency. The fetal imaging signs served as reliable indicators for early diagnosis, especially in the background of consanguinity. We present the cases of the siblings, including a literature review on fetal and neonatal characteristics, as well as the biochemical and molecular features of CPT II deficiency.

## Introduction

**Figure 1:**
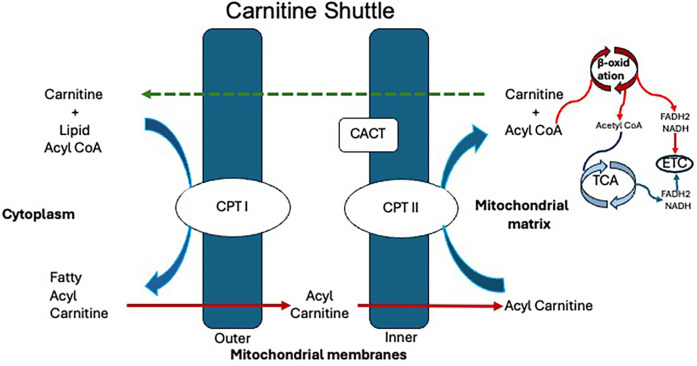
Carnitine shuttle. CACT, carnitine-acylcarnitine translocase; CoA, coenzyme A; CPT I, carnitine palmitoyltransferase I; CPT II, carnitine palmitoyltransferase II; ECT, electron transport chain; FADH2, flavin adenine dinucleotide; NADH, nicotinamide adenine dinucleotide.

Carnitine regulates the balance between free CoA and acyl-CoA both inside and outside the cell. A critical step in fatty acid oxidation is the transport of long-chain fatty acids (LCFAs) into the mitochondria via the carnitine shuttle facilitated by carnitine palmitoyltransferase (CPT) I and II and carnitine-acylcarnitine translocase (CACT), which play a crucial role in energy production. The carnitine shuttle regulates the entry of acyl-CoA esters into the mitochondria. The shuttle requires three major enzymes, namely, CPT I, CACT, and CPT II (1). There are two isoforms of CPT I: Liver (L-CPT I) and the muscle forms (M-CPT I). The lungs, pancreas, ovary, intestine, spleen, and brain also have L-CPT I. In the fasting state, the LCFA are activated in the cytosol of the cytoplasm to form fatty acyl CoA, which then enters the mitochondria. CPT I in the outer mitochondrial membrane facilitates the conversion of acyl-CoA and carnitine to acylcarnitine and free CoA, allowing its entry into the intermembranous space. CPT I is upregulated in the fasting state when low levels of intracellular malonyl-CoA are present. Acylcarnitine is then translocated through the inner mitochondrial membrane in exchange for free carnitine in the presence of transporter CACT. The CPT II enzyme is in the matrix side of the inner mitochondrial membrane, encoded by the *CPT II* gene (chromosome 1p32.3). In the last step, CPT II enzyme activity converts acylcarnitine back to acyl-CoA's, which undergoes β-oxidation, which involves dehydrogenation, hydration, another dehydrogenation, and thiolytic cleavage. Regenerated carnitine is pumped back to the cytosol by CACT to be recycled again in the carnitine shuttle (Figure 1) (1, 2).

CPT II deficiency has three main forms: (1) severe lethal neonatal form (LNF), inherited in an autosomal recessive manner. Affected infants are symptomatic at birth with hypoketotic hypoglycemia, seizures, cardiomyopathy/arrythmias, liver failure and death occurring within the first few weeks of life, (2) severe infantile (hepatocardiomuscular) form, with symptomatology appearing from 6 to 24 months of life in a similar presentation but less severe than LNF form and (3) myopathic form, which is the least severe type and they become symptomatic in infancy to adulthood with myalgia and weakness following exercise or fasting (3–5).

Lethal neonatal form of CPT II remains a challenge in early diagnosis and treatment. We present a consanguineous family who had two children in consecutive pregnancies with LNF presented in a similar way in the fetal and neonatal periods.

## Case presentation

We describe a pair of siblings—an older female (patient A) and a younger male (patient B) with CPT II deficiency born by normal vaginal delivery to a first-degree consanguineous couple who have two healthy male living children. The mother had a history of an early pregnancy loss. The sibling's clinical presentations and investigations were consistent with the severe LNF of CPT II deficiency.

Antenatally noted fetal nephromegaly with renal cysts, cardiomegaly, and ventriculomegaly in both pregnancies. Siblings were born small for gestational age (SGA) and did not require active resuscitation. There was no family history of inborn errors of metabolism (IEM).

Patient A presented with hypothermia and hypoglycemia on day 1 of life while on observation in special care nursery for fetal anomalies. With in hours of stabilization of glucose profile and temperature, baby developed apnea, seizures, intractable arrhythmias, and circulatory collapse. She required full respiratory support. Hyperkalemia and acidosis worsened and was treated accordingly. Following persistent cardiorespiratory failure and intractable ventricular arrythmias she was placed on extracorporeal membrane oxygenation(ECMO) support and hemodialysis. ECMO support was withdrawn after documentation of severe intracranial bleed and succumbed on day 14 of life. The parents were given genetic counseling following the death of patient A.

**Figure 2:**
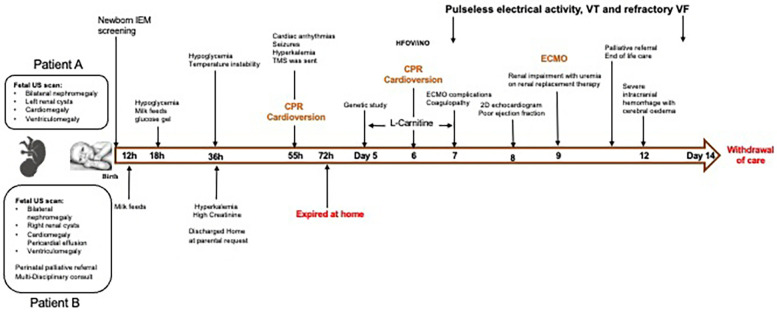
Timeline depicting the sequence of events from fetus to end of life of both siblings. CPR, cardiopulmonary resuscitation; ECMO, extracorporeal membrane oxygenation; HFOV, high frequency oscillatory ventilation; h, hour of age; IEM, inborn errors of metabolism; iNO, inhaled nitric oxide; TMS, tandem mass spectrometry; US, ultrasound; VF, ventricular fibrillation; VT, ventricular tachycardia.

The family declined prenatal diagnostic tests offered in subsequent pregnancy. Given the similar presentation antenatally to patient A, the parents chose to pursue comfort care for patient B and decided against full resuscitation and active medical intervention. Parents agreed to needful investigations and genetic testing at birth. Before the birth of patient B, advanced care planning, including milk bonding and memory making, was offered to parents by the perinatal palliative (PeriPal) care team. The patient was discharged home according to the antenatal plan on day 2, on a special milk feed. The baby passed away on day 3 of life at home with family members around. Figure 2 illustrates a timeline of the fetal and neonatal events of the siblings.

**Figure 3:**
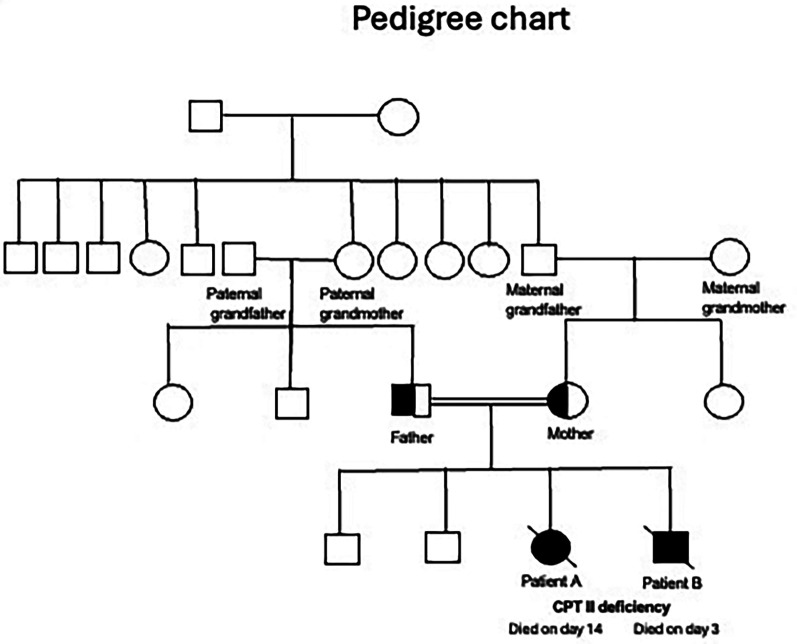
Genogram. CPT II, carnitine palmitoyltransferase II.

A summary of the antenatal and postnatal findings and biochemical and genetic test results of patients A and B are given in Table 1. Table 2 shows the newborn screening results of the siblings. Figure 3 shows the genogram depicting the autosomal recessive inheritance pattern of CPT II deficiency in this family.

**Table 1:** Case description of patient A and patient B.

	Patient A	Patient B
Family history	First-degree consanguineous couple	
	No known medical illness among family members	
Fetal Imaging	Bilateral nephromegaly.	Bilateral nephromegaly
	Echogenic kidneys and renal cysts	Echogenic kidneys and right renal cysts
	Cardiomegaly	Cardiomegaly with pericardial effusion
	Mild ventriculomegaly	Bilateral ventriculomegaly
Multi-disciplinary	No	Yes Palliative team Metabolic team Dietetic team
Involvement prior to delivery		
Birth history	Term (39 + 3 week), SGA, Female	Term (37 week), SGA, Male
	Birth weight 2545 gm	Birth weight 2356 gm
	Normal vaginal delivery	Normal vaginal delivery
Perinatal events	No resuscitation	No resuscitation
Physical examination	No dysmorphic features	No dysmorphic features
Presentation	Hypoglycemia at 6 h of life	Tolerated feeds well
	Temperature instability	Hypoglycemia
	Generalized seizures	Temperature instability
Diagnosis—Biochemical - Genetics	Tandem Mass Spectrometry: Table 2 Molecular testing: (a) Homozygous pathogenic variant, c.63dup (p.Ser22GInfs*37), confirming CPT II deficiency. (b) Homozygous likely pathogenic variant was identified in *SLC22A5* (c.641C > T(p.Ala214val), in keeping with primary carnitine deficiency	Tandem mass spectrometry: Table 2 Molecular testing: Homozygous pathogenic variant in CPTII c.63dup (p.Ser22Glnfs*37)
Other investigations A) Abnormal biochemical analysis: B) 2D Echocardiogram C) Electrocardiogram D) Neonatal Imaging (i.) US Kidneys (ii.) US Brain	Troponin I−50,000 ng/L Serum creatinine kinase- 4,074 U/L CK-MB−295 IU/L ALT−132 IU/L AST−701 IU/L Serum creatinine −210 umol/L Structurally normal heart with biventricular hypertrophy Hyperkalemia with widened QRS complex and tall symmetrical T waves Refractory ventricular fibrillation Bilateral polycystic kidney disease Bilateral ventriculomegaly	Potassium—7.5 mmol/L Creatinine—159 umol/L Urea—8 mmol/L Creatinine kinase—427 U/L Liver function test normal Biventricular hypertrophy Small patent ductus arteriosus Atrial septal aneurysm Normal rhythm Ventriculomegaly Bilateral polycystic kidney disease Mild ventriculomegaly
Outcome	Expired on day 14	Expired on day 3

ALP, alkaline phosphatase; AST, aspartate aminotransferase; CK-MB, creatine kinase-MB; CPT II, deficiency-carnitine palmitoyl transferase II deficiency; SGA, small for gestational age; US, ultrasound.

**Table 2:** Newborn screening panel.

Newborn screening panel	Patient A	Patient B	Reference Units (umol/L)
C0—Free Carnitine	3	13	Abnormal <15
C2—Acetylcarnitine	2.8	2	Abnormal <7
C16- Hexadecanoylcarnitine	9.53	7.97	Abnormal >7
C16:1-Hexadecenoylcarnitine	0.80	2.68	Abnormal >0.23
C18- Octadecanoylcarnitine	3.27	2.13	Abnormal >2.5
C18:1-Octadecanoylcarnitine	4.57	5.30	Abnormal >3
C18:2-Linoleylcarnitine	0.32	1.66	Abnormal >0.16
(C16+C18:1)/C2 ratio	5.03	6.63	Abnormal >0.3

Patient A also had a likely pathogenic variant in *SLC22A5* (p.Ala214Val) indicative of primary carnitine deficiency but needed additional data to prove that conclusively (Invitae, San Francisco, USA). But coexistence with pathogenic *CPT II* gene mutation may explain the more complicated neonatal period of patient A compared to patient B.

## Pathophysiology of CPT II deficiency

The LNF is a multisystemic disease characterized by liver failure with hypoketotic hypo glycemia, cardiomyopathy, arrhythmias, and seizures. Among the CPT II deficiency cases reported, LNF constitutes 6%, infantile form 8%, and adult myopathic form 86% (6). Many *CPT II* gene variants can cause CPT II deficiency. However, only 16 variants were associated with LNF. The residual activity of the CPT II enzyme is very low in neonatal and infantile forms, resulting in abnormal LCFA profiles, notably with high levels of C16 and C18OH (5). This leads to energetic failure due to decreased β-oxidation, thus systemic lipid accumulation. Since the body is unable to use LCFA, it must rely on glucose to generate energy. While unable to metabolize fat, following the depletion of glucose stores, hypoketotic hypoglycemia sets in. Classically, IEM screen in neonates detect markedly abnormal acylcarnitine profile in LNF form but not always in infantile hepato-cardio-muscular form, which often presents later between 6 and 24 months of age (7, 8).

## Clinical and metabolic features

In 1973, DiMauro and DiMauro reported the first case of an adult-onset form of CPT II deficiency (7). We described the presentation of LNF of CPT II deficiency in a family comprising two siblings born to a consanguineous couple. In the following discussion, we review the physiology of carnitine, the antenatal and neonatal presentations, and the diagnosis and management of CPT II deficiency.

Carnitine plays a crucial role in the entry of LCFA via carnitine shuttle into the mitochondrial matrix for β-oxidation and energy production in the form of adenosine triphosphate. Carnitine also provides acetyl-CoA for gluconeogenesis (1). Carnitine cycle defects include (a) primary carnitine deficiency, (b) CPT I deficiency, (c) CACT deficiency, and (d) CPT II deficiency (8). The reported patient A had two homozygous variants: one pathogenic *CPT II* gene, confirming CPT II deficiency and a likely pathogenic variant in *SLC22A5* gene indicating primary carnitine deficiency (Table 1).

Antenatal phenotypes include facial dysmorphism, cerebral malformations, cystic dysplastic kidneys, cardiomegaly, and diffuse fatty infiltration (9). After reviewing 19 cases, Boemer et al. found that the malformations associated with CPT II deficiency include renal cysts/nephromegaly in 57%, cardiomegaly in 15%, and severe cerebral dysgenesis in 74%. Cerebral malformations observed included neuronal migration disorders, such as polymicrogyria and pachygyria, cystic dysplasia of the brain, cerebellar vermian hypoplasia, hydrocephalus, and agenesis of the corpus callosum (10). Maternal liberal fetal supply of glucose as a substrate leads to high concentrations of malonyl-CoA, which inhibits CPT I. The entry of LCFA via carnitine shuttle into the mitochondria is inhibited with low CPT I activity, impairing fatty acid oxidation (11). Oey et al. demonstrated high CPT II enzyme activity in the heart, liver, and brain in a 6-week-old embryo, explaining multiorgan involvement in CPT II deficiency (12). This was reflected in our siblings who presented antenatally with polycystic kidneys, ventriculomegaly, and cardiomegaly, showing that these clinical features in fetal scans could be an indicator of CPT II deficiency, particularly in cases involving consanguineous couples. In cases with a known family history, parents may be offered pre-implantation genetic diagnosis for future pregnancies (13, 14). Unfortunately, most fetuses with major malformations-particularly involving the brain and kidneys, are terminated, resulting in missed opportunities for metabolic/genetic screening.

The LNF of CPT II deficiency is often symptomatic shortly after birth. The affected infant may exhibit dysmorphic features, including microcephaly, overfolded helices, a sloping forehead, long and tapered fingers and toes, contractures, and hypoplastic toenails (15); however, none were observed in our case studies. Other manifestations of this multisystemic disease include cardiac arrhythmias, cystic dysplastic kidney, cardiomyopathy, and hypoketotic hypoglycemia (10, 16). Structurally abnormal brain and neuronal migration defects can result from defective β-oxidation, leading to impaired phospholipid synthesis and accumulation of toxic metabolites (9, 17). Both of our patients developed hypoglycemia as the presenting feature. Patient A experienced intractable seizures, cardiovascular collapse following recurrent arrhythmias, and severe hyperkalemia in the immediate newborn period and died at 2 weeks of age despite maximum optimal therapy. For the younger child, on fetal detection of renal and cardiac defects, parents were counseled towards comfort care if confirmed CPT II deficiency postnatally with biochemical profiling. Due to the early discharge of patient B, other life-threatening symptoms were not observed.

Among the various phenotypic forms of CPT II deficiency, the severe infantile hepatocardiomuscular type typically presents with hypoketotic hypoglycemia, liver failure, and cardiomyopathy within the first year of life. It can be fatal, even with early intervention. The more common myopathic form is found in adults, and symptoms of muscle pain and weakness usually appear during periods of fasting or with intercurrent illness (8, 18).

## Diagnosis

The use of tandem mass spectrometry (MS/MS) is common in neonatal metabolic screening. Significant elevations of C16 and C18-acylcarnitine with decreased free carnitine indicate CPT II deficiency. Total and free carnitine levels are often reduced with higher-than-normal levels of acylcarnitine so that the acylcarnitine to free carnitine ratio is increased (19, 20). For a definitive diagnosis, rapid molecular genetic testing is recommended. Pathogenic variants of the *CPT II* gene, which cause either truncation of the protein or degradation of its *mRNA,* are associated with LNF of CPT II deficiency (8, 21). Mutation analysis can be done on fibroblasts, blood, skeletal muscle, and lymphoblasts (22–24). In LNF and infantile forms, intrafamilial phenotypic homogeneity is a known feature. Still, the prediction of the phenotype from prenatal tests remains scarce, and the genotype-phenotype correlation remains imprecise (21, 25).

Alternatively, the diagnosis can be made by detecting decreased CPT II enzyme activity in cultured fibroblasts (10). In LNF, CPT II activity in cultured fibroblast cells is 7%, whereas the enzyme activity is 2.5%–10% in infantile and 20%–46% in the milder adult myopathic forms. Liver function is often deranged with low prothrombin activity. Raised serum creatine kinase and transaminitis of 20–400 times higher have been observed biochemically (26). Prenatal and pre-implantation genetic testing can be offered to parents who are carriers of CPT II deficiency for future pregnancies (13). In both infants, molecular testing identified a homozygous pathogenic variant in *CPT II* c.63dup (p.Ser 22Glnfs*37), inherited from both parents. This sequence change creates a premature translational stop signal in the *CPT II* gene and is expected to result in an absent or disrupted protein product. Loss-of-function variants in the *CPT II* gene are known to be pathogenic (8).

## Management

Suspect IEM in infants born to consanguineous couple with bad obstetric history or fetal brain/cardiac and/or renal anomalies. Early symptomatology in a neonate like lethargy, vomiting, poor suck, and disorientation should raise concern of IEM. At birth, dextrose infusion is recommended to slow down catabolism, helping to prevent recurrent hypoglycemia, awaiting tandem mass spectrometry report. Once abnormal acylcarnitine profile is detected, treatment focuses on nutrition with carbohydrate-rich (70%) and low-fat (<20%) feeds, incorporating medium-chain triglycerides to support glycolysis. Triheptanoin, a medium-chain triglyceride, is used in the treatment of fatty acid oxidation disorders (27). It provides a source of calories and fats needed for energy production. Studies have shown that triheptanoin reduces the likelihood of patients developing hypoglycemia, cardiomyopathy, rhabdomyolysis, and hepatomegaly (28). Despite the initiation of triheptanoin supplementation and a high-carbohydrate diet, our patient B passed away shortly thereafter, being an LNF of CPT II deficiency. The lack of active resuscitative efforts due to comfort care measures may also have contributed to the outcome in patient B.

Carnitine supplementation is a common practice in IEM, especially in primary and secondary carnitine deficiency. Carnitine has been used in the treatment of CPT II deficiency; however, its use remains controversial. It is intended to convert potentially toxic long-chain acyl-CoA compounds into acylcarnitine, which can then be excreted in the urine, depleting free CoA in the mitochondria (16, 29). In mouse models, acylcarnitine has been shown to regulate the hERG (human ether-a-go-go related gene) channel, which is crucial for cardiac repolarization and may contribute to the development of arrhythmias (30, 31). In the case of patient A, initiation of carnitine supplementation was associated with refractory arrhythmias, leading to discontinuation of the treatment. However, a Cochrane review found insufficient evidence to support the efficacy and safety of carnitine supplementation in IEM (32).

It is also important to avoid known triggers, including extreme temperatures, illness, and medications such as sodium valproate, diazepam, and ibuprofen (9, 33, 34). Frequent feedings are advised to prevent fasting, along with the administration of carnitine supplements (3, 8). In patients with cystic dysplastic kidney disease, maintaining adequate hydration is crucial to slow the progression to renal failure (35).

Bezafibrate, a class of hypolipidemic drugs, increases *CPT II* mRNA and normalizes enzyme activity in mild forms of CPT II deficient myoblasts. In a trial of fibrates in adult myopathic forms, muscle biopsy specimens showed a marked increase in palmitoyl L-carnitine oxidation levels by 60%–284% and *CPT II* mRNA levels by 20%–93%. Clinically, reduced episodes of rhabdomyolysis with significant improvement in quality of life over 6 months of treatment (36).

Advancements in antenatal diagnostic techniques have enabled the detection of a range of fetal anomalies of varying severity in CPT II deficiency. In some cases, treatment options may be limited or unavailable, or the condition may be potentially fatal, leading to neonatal death. The establishment of a structured program that includes PeriPal care alongside neonatologists could offer comprehensive support for vulnerable babies with life-limiting conditions. If parents choose to continue with the pregnancy, early counseling by the PeriPal team is beneficial to help alleviate parental anxiety, ensure clear, consistent communication, and make advance care plans for the baby (35). The team will collaborate with community palliative care (Star PALS team) services to ensure the transition of care from hospital to home, provide a comprehensive plan through home visits, and offer psychosocial support. In the event of demise, the team will also provide bereavement support.

## Genetic counselling

CPT II deficiency is an autosomal recessive disorder with a probability of 25% being affected and 50% being carriers in future pregnancies for a consanguineous couple who are carriers for this variant. Molecular genetic testing for the *CPT II* gene or CPT II enzyme activity assay can give a prenatal diagnosis in pregnancies at increased risk for mild to severe forms of CPT II deficiency. To date, 16 variants have been reported in 26 lethal neonatal cases, born to 11 different families (5). Our reported cases had homozygous pathogenic variants in *CPT II*—c.63dup (p.Ser22GInfs*37). Patient A also had a homozygous likely pathogenic variant- *SLC22A5* (c.641C>T(p.Ala214val), indicative of a diagnosis of primary carnitine deficiency. El-Hattab et al. reported the variability in presentation of systemic primary carnitine deficiency from metabolic decompensation in infancy to an asymptomatic adult (37).

## Conclusion

Antenatal detection of fetal anomalies are reliable indicators for suspecting CPT II deficiency in the background of parental consanguinity, more so if a previous baby was affected. In a suspected case of CPT II deficiency the following are considered: (1) dextrose infusion to prevent catabolism till special milk feeds can be arranged, (2) a high carbohydrate and low-fat diet to provide substrate for glycolysis, (3) avoidance of known triggers, e.g., fasting, illness etc., (4) urgent IEM screen/genetic testing/echocardiography/and ultrasound scan of the brain and kidneys, (5) MCT supplementation, (6) intensive care monitoring, and (7) a multidisciplinary team including metabolic specialists and palliative care doctors. As the neonatal form of CPT II deficiency is typically fatal, early confirmation of the diagnosis should prompt advance care planning discussions with the parents to support timely, appropriate care and informed decision-making in the best interest of the child.

## Patient's perspective

“The family was in shock after seeing our daughters struggle to survive. For the son, we were strong enough to decide on comfort care with the timely support of the palliative team.”

## Data Availability

The original contributions presented in the study are included in the article/Supplementary Material, further inquiries can be directed to the corresponding author.
